# Simultaneous Stenting for Symptomatic Tandem Extracranial and Intracranial Posterior Circulation Stenoses: Long-Term Outcomes and Procedural Experience

**DOI:** 10.3389/fneur.2021.724985

**Published:** 2021-08-31

**Authors:** Zichang Jia, Yanqing Zhao, Peng Wang, Jintao Han, Shilu Zhao, Xuan Li

**Affiliations:** ^1^Department of Interventional Radiology and Vascular Surgery, Peking University Third Hospital, Beijing, China; ^2^Department of Neurology, The Third People's Hospital of Liaocheng, Liaocheng, China

**Keywords:** stenting, posterior circulation, long-term, stenoses, tandem

## Abstract

Few studies have reported on simultaneous endovascular stenting for tandem posterior circulation (PC) stenoses and its long-term outcomes. Thus, our aim was to investigate the safety and efficacy of simultaneous stenting in patients with symptomatic tandem extra- and intracranial PC stenoses. From September 2014 to June 2018, 16 such patients with symptomatic stenoses who underwent simultaneous stent placement were analyzed. The primary outcome was occurrence of any stroke, TIA, or death within 30 days after the procedure. The secondary outcomes were technical success, clinical success, and the occurrence of in-stent restenosis ≥50% during follow-up. Technical success was defined as stent coverage of all tandem lesions and residual stenosis <30%. Clinical success was determined based on any occurrence of neurological events or death within 3 months after the procedure. All stents (19 intracranial and 14 extracranial) were placed with a technical success rate of 100%. One patient experienced a pontine ischemic stroke 2 days after the procedure and had recovered well at discharge. One patient experienced a minor complication of groin hematoma. The clinical success rate was 93.75% (15/16). During a median follow-up of 36.0 ± 11.0 months, two patients developed ISR ≥50% at the 1-year follow-up. None of the patients experienced stroke, TIA, or death after discharge during follow-up. Simultaneous stenting for symptomatic tandem extra- and intracranial PC stenoses is safe and feasible. Its impact on long-term stroke prevention is promising, and further study of a larger patient population is needed.

## Introduction

Posterior circulation (PC) strokes account for nearly 30% of all ischemic strokes, the main etiology of which is atherosclerotic vertebrobasilar disease (1–3). The annual risk of recurrent stroke or sudden death in patients with symptomatic vertebrobasilar stenosis may reach 10.9–25.5% despite optimal medical therapy (4–7). Patients with severe (≥70%) intracranial stenosis, especially coexisting tandem extra- and intracranial stenoses, have grimmer outcomes than those with no or mild stenosis (3, 8, 9).

To date, various randomized clinical trials have demonstrated the safety and feasibility of endovascular stenting for patients with either extra- or intracranial PC stenosis (10–12). However, there are few reports of endovascular stenting for tandem extra- and intracranial PC stenoses (13, 14). Moreover, the techniques and long-term outcomes of simultaneous stenting for tandem extra- and intracranial PC stenoses are rarely reported. Thus, this study aimed to investigate the safety and efficacy of simultaneous stenting in patients with symptomatic tandem extra- and intracranial PC stenoses.

## Materials and Methods

### Study Design

This retrospective study was approved by our institutional review board, and informed consent was obtained from all the patients. A neurointerventional patient database was reviewed to identify eligible patients treated at our institution between September 2014 and June 2018. The inclusion criteria were as follows: (a) patients who experienced a PC transient ischemic attack (TIA) or were in chronic stage of PC ischemic stroke; (b) recurrent ischemic events in the territory of the stenosed artery despite optimal medical treatment; (c) occluded or hypoplastic contralateral vertebral artery and poor intracranial collaterals; (d) simultaneous stent placement in patients with severe (≥70%) tandem stenoses (≥2 lesions, but not contiguous) in the ipsilateral extracranial vertebral artery and intracranial vertebrobasilar artery (VBA); (e) modified Rankin Scale (mRS) score ≤ 2. We excluded patients with VBA stenosis caused by dissection, fibromuscular dysplasia, vasculitis, and all other non-atherosclerotic etiologies.

The National Institutes of Health Stroke Scale (NIHSS) score was used by a stroke neurologist to evaluate neurological deficits at admission and discharge. To assess the degree of disability and clinical outcome, the mRS score was measured for each patient at admission, 3 months after discharge, and at the last follow-up. The tandem PC stenoses and arterial anatomy were initially assessed by computed tomography angiography or magnetic resonance angiography and further validated by digital subtraction angiography (DSA). Extracranial stenosis of the vertebral artery was quantified using the NASCET angiographic measurement method (15). Stenosis of the intracranial VBA was quantified and characterized according to the WASID angiographic measurement method (16). Mori's angiographic classification (type A: short concentric, ≤ 5 mm, moderate to severe stenosis with a smooth contour and non-tortuous proximal anatomy; type B: longer, <10 mm, eccentric severe stenosis with an irregular contour and slightly tortuous proximal anatomy; and type C: long, >10 mm, eccentric severe stenosis with an irregular contour and highly tortuous proximal anatomy) was used to assess the intracranial lesion characteristics (17). The medical records and imaging studies were reviewed to collect data on patient demographics and clinical characteristics, technical success, clinical outcomes, complications, and survival.

### Endovascular Technique

All procedures were performed under general anesthesia with endotracheal intubation. A 6F sheath was introduced into the femoral (*n* = 14) or radial (*n* = 2) artery. A 90-cm 6F guiding catheter was passed through the sheath and positioned in the subclavian artery close to the VAO. In cases of severe VAO stenosis, pre-dilation of the stenosed ostium was first performed with a 3- or 4-mm balloon catheter without an embolic protective device. The guiding catheter was then navigated into the distal second segment of the vertebral artery with or without the aid of a balloon catheter. After confirming smooth antegrade flow in the VBA, a 0.014-inch micro-guidewire was delivered to cross the stenotic intracranial VBA lesion under roadmap fluoroscopic guidance to avoid vessel damage. A balloon catheter (Gateway, Stryker, Michigan, USA) and stent delivery device (Apollo stent, Microport, Shanghai, China; Wingspan stent, Stryker, Michigan, USA) were then delivered over the micro-guidewire and navigated to the stenotic lesion. The total length of the stent should cover and extend past the lesion by ~2 mm at both ends. The diameter should be slightly less than that of the normal adjacent lumen (0.8–0.9:1.0) in the intracranial VBA or vertebral artery lesion.

After placement of the intracranial stent, the guiding catheter was withdrawn from the subclavian artery to perform VAO stenting (Express Vascular SD stent, Boston Scientific, Massachusetts, USA). The diameter of the deployed stent was the same or slightly larger than that of the normal lumen (1–1.1:1) in the VAO lesion. After the stenting procedure, a final angiography was performed to evaluate the residual stenosis and intracranial blood flow. Repeated balloon angioplasty was performed in the same session if the residual stenosis was >30%. During the procedure, all catheters were connected to a continuous heparinized saline flush (3,000 U/500 mL).

### Periprocedural Management and Follow-Up

Aspirin (100 mg/day) and clopidogrel (75 mg/day) were administered to all patients for at least 5 days before the procedure. Nimodipine was intravenously infused for 2 h before the procedure to prevent vasospasm. After the procedure, non-enhanced computed tomography was routinely performed to exclude intracranial hemorrhage. The combination of aspirin (100 mg/day) and clopidogrel (75 mg/day) was administered for at least 3 months after the procedure, followed by long-term monotherapy with either aspirin or clopidogrel according to the thromboelastogram results.

Follow-up visits with neck Doppler ultrasound were scheduled at 1 month and 3 months after the procedure and every 6 months thereafter until death. Computed tomography or magnetic resonance imaging were immediately performed in patients who developed neurological symptoms. DSA or computed tomography angiography was recommended for all patients at the 1-year follow-up after the procedure. The mRS score of each patient after the procedure was obtained at a clinic visit or telephone interview.

### Outcomes and Definitions

The primary outcome was the safety of the procedure, including the occurrence of any stroke, TIA, or death within 30 days after the procedure. Stroke and TIA were defined as in previous studies and were assessed by neurologists (11, 18). The secondary outcomes were technical success, clinical success, and the occurrence of in-stent restenosis (ISR) ≥50% during follow-up. Technical success was defined as complete stent coverage of all tandem lesions and residual stenosis <30%. Clinical success was determined based on the occurrence of any neurological events or death within 3 months after the procedure. Periprocedural complications were defined as all-cause morbidity and mortality within 30 days after the procedure. Major complications included stroke and death.

### Statistical Analysis

Continuous data are expressed as mean ± SD, whereas categorical data are reported as numbers and percentages. Comparisons between pre- and post-procedure variables were performed using the Wilcoxon rank-sum test for numerical values and Fisher's exact test for categorical values. A two-sided *P*-value of ≤ 0.05 was considered statistically significant. All statistical analyses were performed using SPSS software (IBM SPSS Statistics 25, Chicago, IL, USA).

## Results

A total of 132 patients with PC stenosis were treated at our institution between September 2014 and June 2018. During the study period, 17 patients were initially selected. One patient was excluded because of a non-atherosclerotic etiology (arterial dissection). A total of 16 patients (10 male; mean age, 66.6 ± 8.1 years) were included in the final analysis. Among them, 11 patients experienced posterior ischemic stroke and 5 patients experienced TIA. The average NIHSS score at admission was 1.3 ± 1.0 (range, 0–3). The average mRS score at admission was 0.3 ± 0.4 (range, 0–1). The details of demographic and clinical characteristics are summarized in Table 1.

**Table 1:** Demographics and clinical characteristics of the 16 patients.

**Variables**	**Number (%)**
Age, y	66.6 ± 8.1
Male	10 (62.5)
Risk factors	
Hypertension	11 (68.8)
Hyperlipidemia	8 (50.0)
Diabetes mellitus	7 (43.7)
Smoking	7 (43.7)
Qualifying events	
Ischemic stroke	11 (68.8)
TIA	5 (31.2)
NIHSS score at admission	1.3 ± 1.0
0	5 (31.3)
1	2 (12.5)
2	8 (50.0)
3	1 (6.25)
mRS score at admission	0.3 ± 0.4
0	12 (75.0)
1	4 (25.0)
Contralateral VA condition	
Occluded	7 (43.7)
Hypoplastic	9 (56.3)
Tandem stenotic sites	
Ipsilateral VAO + V4	8 (50.0)
Ipsilateral VAO + BA	5 (31.3)
V4 + BA	2 (12.5)
Ipsilateral VAO + V4 + BA	1 (6.3)
Mori type of the intracranial lesions	
Mori A	12 (63.2)
Mori B	5 (26.3)
Mori C	2 (10.5)
Stent type for intracranial lesions	
Apollo stent	10 (52.6)
Wingspan stent	9 (47.4)

*Data are presented as mean ± standard deviation or as frequency (percentage)*.

*TIA, transient ischemic attack; NIHSS, National Institutes of Health Stroke Scale; mRS, modified Rankin Scale; VA, vertebral artery; VAO, vertebral artery ostium; V4, fourth segment of vertebral artery; BA, basilar artery*.

**Figure 1:**
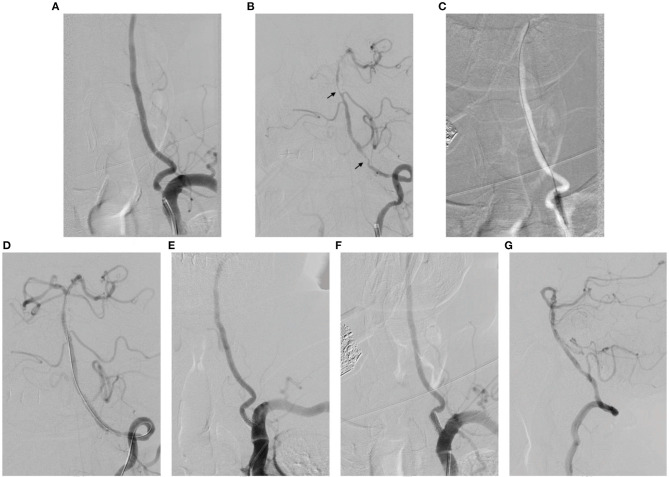
A 76-year-old man with symptomatic tandem extra- and intracranial posterior circulation stenoses underwent simultaneous stent placement. **(A, B)** Pre-procedural digital subtraction angiography (DSA) showed severe tandem stenoses at the origin of the left vertebral artery (VA), V4 segment artery, and basilar artery (BA) (arrows) and insufficient posterior cerebral blood flow. **(C)** A 4–20-mm angioplasty balloon was used to dilate the origin of the left VA. **(D)** Stenting was performed with 2.5–13 and 3–13-mm balloon-mounted stents within the BA and V4 segment artery, respectively. **(E)** Finally, a 5–15-mm balloon-mounted stent was placed at the VA origin. **(F, G)** DSA at the 12-month follow-up of this patient showed no obvious in-stent restenosis. There was no recurrence of ischemic events during the follow-up.

Sixteen patients underwent 16 simultaneous stenting procedures for tandem PC stenoses with 33 stents. All stents were placed successfully and covered all the lesions without residual stenosis >30%, rendering a technical success rate of 100% (33/33) (Figure 1). One patient (6.3%) experienced limb weakness and dizziness (NIHSS score = 4) 2 days after basilar stent placement. On post-procedure MRI, pontine ischemic stroke was confirmed in this patient. After treatment with antiplatelet agents and intravenous hypervolemic hemodilution, this patient showed good recovery at discharge (NIHSS score = 1; mRS = 0) and at the 3-month follow-up (mRS = 0). One patient (6.3%) experienced a minor complication of groin hematoma. No neurological events or deaths were encountered in the remaining patients within 3 months after the procedure. Therefore, the clinical success rate was 93.75% (15/16). The NIHSS scores at admission and discharge were not significantly different among the 16 patients (1.3 ± 1.0 vs. 1.0 ± 0.8, respectively; *P* = 0.327).

Patients were followed for a median of 36.0 ± 11.0 months (range, 18.0–62.0 months), and two patients were lost after 18 and 22 months follow-up, respectively. At the 1-year follow-up, DSA was performed in 10 patients (Figure 1). ISR ≥50% was confirmed in two patients (12.5%, 2/16; ISR sites, VAO and BA, respectively) and both were asymptomatic; therefore, no intervention was needed. None of the patients experienced stroke, TIA, or death during follow-up after discharge. No significant changes were found in the mRS scores [at admission vs. 3-month follow-up, 0.3 ± 0.1 vs. 0.3 ± 0.1 [*P* = 1.000]; at admission vs. last follow-up, 0.3 + 0.1 vs. 0.2 ± 0.1 [*P* = 0.681]].

## Discussion

In contrast to ischemic carotid artery territory events, which have been extensively investigated due to the development of carotid endarterectomy and endovascular stenting, the PC TIA or stroke has largely been overlooked (19). Despite the best medical therapy, symptomatic atherosclerotic vertebrobasilar stenosis is associated with a high risk of recurrent stroke and sudden death (4–7). Two prospective studies—one hospital-based study in 216 patients and one population-based study in 151 patients—showed that the risk of recurrent PC stroke was 30.5% in patients with ≥50% stenosis vs. 8.9% in patients without such stenosis, and VBA stenosis ≥50% was associated with 3.2-fold higher risk of recurrent ischemic PC events within 90 days, reaching 22% for stroke and 46% for TIA and stroke (7, 20). Moreover, Wong et al. documented the long-term outcomes of 705 ischemic stroke patients, of whom 345 had large-artery lesions. The outcomes were grimmer for patients with tandem extra- and intracranial stenoses, reaching a risk of death, cerebrovascular events, or recurrent stroke within 1 year of 24.3% (8). The optimal management of tandem extra- and intracranial stenoses, however, is a matter of ongoing debate, and high-level evidence and guidelines are still lacking.

Endovascular angioplasty and stenting in the PC are now technically feasible, whereas endovascular therapies for coexisting tandem extra- and intracranial PC stenoses remain limited. Furthermore, stent placement for tandem intracranial and VAO lesions is technically more complicated than for an isolated lesion (13). Du et al. reported that technical success (complete stent coverage of the lesion and residual stenosis <30%) was achieved in nine of 10 patients who underwent stenting for tandem stenoses of the intracranial VBA and VAO. No periprocedural complications were noted, and the annual stroke rate in the VBA territory after stenting was 3.8% (13). In a retrospective study of 16 patients with 27 complex stenotic PC lesions, including 13 patients with tandem lesions, Wang et al. reported that the technical success rate (complete stent coverage of the lesion and residual stenosis <50%) was 100%, with a 12.5% major complication rate (14). Compared to the endovascular techniques of Du et al. and Wang et al., we performed pre-dilation of the stenosed ostium and then navigated the guiding catheter into the distal second segment of the vertebral artery with or without the aid of a balloon catheter (a partially inflated balloon within the VAO may act as a flexibly shaped “cushion” to help steer the guiding catheter into the vertebral artery). After successful placement of the intracranial stent, the guiding catheter was withdrawn into the subclavian artery to perform VAO stenting. In the current study, technical success was excellent (100%, 33/33) and clinical success was high (93.8%, 15/16). One potential key factor for excellent technical success in the present study could be the pre-dilation of VAO lesions and the use of a balloon catheter to advance the guiding catheter through the VAO into the vertebral artery. For patients in whom advancing the guiding catheter into the vertebral artery was difficult, we placed the guiding catheter close to the VAO and stably placed the micro-guidewire at the P2 segment of the posterior cerebral artery to facilitate intracranial stent placement. Another key factor could be the intracranial lesion characteristics. Most intracranial lesions (89.5%) in the present study were Mori type A and B, which have lower technical difficulty and procedural risk.

Angioplasty and stent placement for intracranial stenosis are associated with a higher complication rate than those with extracranial stenosis (11, 21). Markus et al. reported a 15.4% (2/13) stroke rate during intracranial stenting in the Vertebral Artery Ischemia Stenting Trial (VIST); however, no (0/48) periprocedural complications occurred with extracranial stenting (11). Seifert et al. reported a major complication rate of 11.8% (2/17) in patients with vertebrobasilar intracranial stenosis who underwent angioplasty and stent placement. Both patients had tandem lesions in the BA and vertebrobasilar junction (21). Concerning the stenting of tandem stenoses in the PC, Du et al. showed excellent results of zero strokes or deaths within 30 days (13). In this study, one (6.3%) major periprocedural complication was noted after basilar stent placement. After prompt and proper treatment, the patient recovered well after discharge.

This study had several limitations. First, this was a retrospective study; therefore, selective bias inevitably existed. Second, the single cohort study design and small sample size were not feasible to perform statistically univariate or multivariate analyses. However, we provided a median 36-month follow-up of all patients, and 10 (62.5%) underwent repeated DSA for treatment evaluation at a 1-year follow-up.

In conclusion, simultaneous stenting for symptomatic coexisting tandem extra- and intracranial PC stenoses is safe and feasible, with a 6.3% periprocedural stroke risk. Its impact on long-term stroke prevention is promising, and further study of a larger population is warranted.

## Data Availability Statement

The original contributions presented in the study are included in the article/supplementary material, further inquiries can be directed to the corresponding author.

## Ethics Statement

The studies involving human participants were reviewed and approved by Peking University Third Hospital. The patients/participants provided their written informed consent to participate in this study. Written informed consent was obtained from the individual(s) for the publication of any potentially identifiable images or data included in this article.

## Author Contributions

ZJ and YZ contributed equally to drafting the initial manuscript. PW, JH, XL, and SZ contributed to study design and data collection. JH and YZ contributed to manuscript revision. All authors contributed to the article and approved the submitted version.

## Conflict of Interest

The authors declare that the research was conducted in the absence of any commercial or financial relationships that could be construed as a potential conflict of interest.

## Publisher's Note

All claims expressed in this article are solely those of the authors and do not necessarily represent those of their affiliated organizations, or those of the publisher, the editors and the reviewers. Any product that may be evaluated in this article, or claim that may be made by its manufacturer, is not guaranteed or endorsed by the publisher.
